# Finite-Element Modeling of Viscoelastic Cells During High-Frequency Cyclic Strain

**DOI:** 10.3390/jfb3010209

**Published:** 2012-03-22

**Authors:** Jaques S. Milner, Matthew W. Grol, Kim L. Beaucage, S. Jeffrey Dixon, David W. Holdsworth

**Affiliations:** 1 Imaging Research Laboratory, Robarts Research Institute, Schulich School of Medicine & Dentistry, The University of Western Ontario, London, ON N6A 5K8, Canada; E-Mail: milnejs@imaging.robarts.ca; 2 Department of Anatomy and Cell Biology, Schulich School of Medicine & Dentistry, The University of Western Ontario, London, ON N6A 5C1, Canada; E-Mail: matthew.grol@schulich.uwo.ca; 3 Department of Physiology and Pharmacology, Schulich School of Medicine & Dentistry, The University of Western Ontario, London, ON N6A 5C1, Canada; E-Mails: kim.beaucage@schulich.uwo.ca (K.L.B.); jeff.dixon@schulich.uwo.ca (J.S.D.); 4 Department of Surgery, Schulich School of Medicine & Dentistry, The University of Western Ontario, London, ON N6A 4V2, Canada

**Keywords:** mechanotransduction, finite-element modeling, single-cell model, cyclic strain, viscoelasticity, cytoskeleton, stress fibers

## Abstract

Mechanotransduction refers to the mechanisms by which cells sense and respond to local loads and forces. The process of mechanotransduction plays an important role both in maintaining tissue viability and in remodeling to repair damage; moreover, it may be involved in the initiation and progression of diseases such as osteoarthritis and osteoporosis. An understanding of the mechanisms by which cells respond to surrounding tissue matrices or artificial biomaterials is crucial in regenerative medicine and in influencing cellular differentiation. Recent studies have shown that some cells may be most sensitive to low-amplitude, high-frequency (*i.e.*, 1–100 Hz) mechanical stimulation. Advances in finite-element modeling have made it possible to simulate high-frequency mechanical loading of cells. We have developed a viscoelastic finite-element model of an osteoblastic cell (including cytoskeletal actin stress fibers), attached to an elastomeric membrane undergoing cyclic isotropic radial strain with a peak value of 1,000 µstrain. The results indicate that cells experience significant stress and strain amplification when undergoing high-frequency strain, with peak values of cytoplasmic strain five times higher at 45 Hz than at 1 Hz, and peak Von Mises stress in the nucleus increased by a factor of two. Focal stress and strain amplification in cells undergoing high-frequency mechanical stimulation may play an important role in mechanotransduction.

## 1. Introduction

Mechanotransduction plays an essential role in normal cell function and in a wide range of disease conditions [1,2,3]. In addition to the role that mechanotransduction plays in skeletal remodeling in response to weight-bearing loads [4,5], the interaction between cells and the local mechanical environment is critical to the normal functioning of blood vessels [6,7,8,9], heart valves [10,11], lung airways [12,13,14], and renal flow [15,16]. It is also thought that mechanical signals may be important in tumorigenesis and metastasis [2,17,18], and in stem cell differentiation [19,20,21]. However, the specific mechanisms by which cells sense and respond to local mechanical signals are not well understood.

To improve our understanding of how cells interact mechanically with surrounding materials and various substrates, experimental studies are typically carried out with cultured cells or tissues, cyclically stimulated in specially designed bioreactors [22,23,24,25,26]. These investigations may involve cyclic stimulation with oscillating shear in microfluidic chambers [27,28,29,30], cyclic compression [31,32], vibration [24,33,34], or cyclic strain [23,35]. Strain is commonly introduced by stretching cell monolayers on flexible membranes, introducing either uniaxial [36,37,38,39], biaxial [40,41,42,43], or equibiaxial (*i.e.*, radially isotropic) sinusoidally varying strain [36,44,45,46], at frequencies ranging up to a few Hz. Experiments using both cultured cells and small-animal models have indicated that the specific frequency of oscillation may be an important parameter, with frequencies ranging from 10 to 90 Hz recently observed to stimulate significant changes in bone formation and body composition [33,47,48,49,50,51,52,53,54].

In the past several years, advances in finite-element modeling (FEM) capacity have made it possible to model many aspects of cell mechanics [55,56,57,58], including the response to dynamic external mechanical stimuli [56,59,60,61]. It is now possible—using commercially available FEM solvers—to simulate the three-dimensional structure of cells, including the cytoskeleton and nucleus [58,62,63]. With geometric boundary conditions determined from confocal microscopy and material properties estimated from experiments using atomic force microscopy [64,65,66], micropipette manipulation [67,68], or magnetic beads [69], it is feasible to simulate the deformation, stress, and strain response of cells to specific mechanical stimuli. Previous studies have shown that the stress and strain manifested within an adherent cell may be regionally concentrated, resulting in peak values that significantly exceed the magnitude of the applied stimuli [70,71,72].

We report the development of a three-dimensional finite-element model of cyclic strain, simulating adherent osteoblastic cell geometry on an isotropic equibiaxial strain membrane. This model incorporates the viscoelastic properties of the cytoplasm—as well as a basic model of the cytoskeleton—and simulations include sinusoidal strain at frequencies ranging from 1 to 45 Hz, consistent with the range of frequencies used in recent cell and animal experiments. This model is used to investigate the influence of the frequency of mechanical stimulation on peak stress and strain distributions within the cytoplasm and nucleus.

## 2. Experimental Section

### 2.1. Generation of Cell Model Geometry

**Figure 1:**
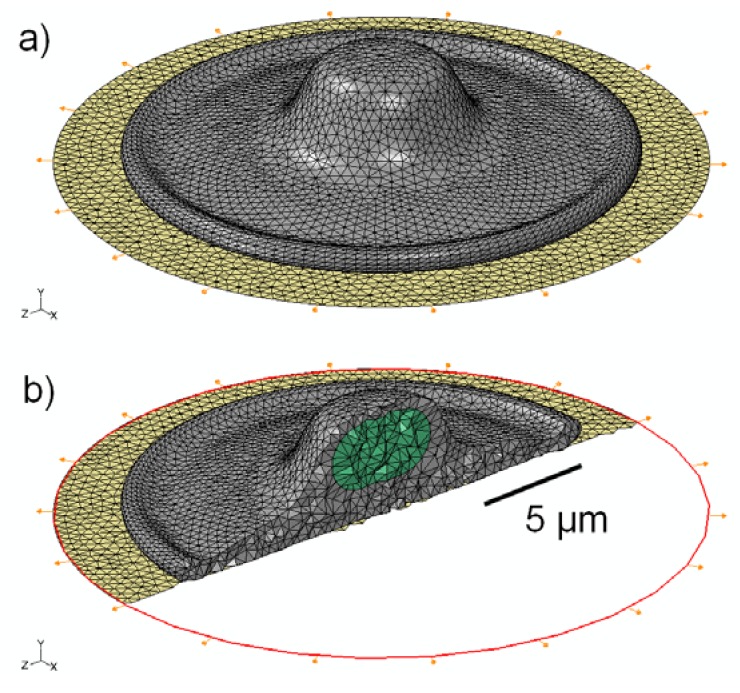
(**a**) Schematic representation of the 3D model geometry used to represent an osteoblastic cell (grey), attached to an elastomeric substrate (gold); (**b**) Cross-sectional view of the 3D mesh, showing the location and shape of the nucleus (green) within the cytoplasm.

The cell geometry used in this study is representative of an adherent osteoblastic cell as it would be configured *in vitro* on a flat substrate. The shape (illustrated in Figure 1) is a rotationally symmetric flattened shell, with characteristic diameter of 20 µm and maximum height of 5 µm. The thinnest section of the “foot” (*i.e.*, the flattened part of the plated cytoplasm) is 1 µm thick and total cell volume is 490 µm^3^. The nucleus is modeled as an ellipsoid, with maximum diameter of 5 µm, minimum diameter of 2 µm and volume of 46 µm^3^. These dimensions are representative of osteoblastic cells observed with confocal microscopy, and are consistent with similar finite-element models reported previously [57,58,73,74].

The cell model is attached to a thin elastomeric membrane, simulating an experimental configuration that would be used to investigate the effect of cyclic strain. The membrane was specified as a 0.25 mm thick sheet of polydimethylsiloxane (PDMS), with a linear elastic modulus of 1.8 MPa and ν = 0.49, where ν is Poisson’s ratio [75]. To simulate physiological strain levels, the sheet was specified to undergo cyclic sinusoidal radial strain with a maximum value of 1,000 µstrain. This produced a radially isotropic (equibiaxial) tensor strain field, centered directly underneath the cell. The cell was assumed to be in contact with the membrane uniformly, with no-slip conditions of adhesion over the entire contact area.

### 2.2. Assignment of Material Properties

Constitute components considered in this finite-element model were limited to the cytoplasm and nucleus. The material properties of the cell were representative of previous experimental studies [76] and similar to values used in previous finite-element models [57,58,73,77,78]. The Generalized Maxwell model is the most commonly employed model for linear viscoelasticity, and has been used successfully in previous FEM studies of cells [62,79,80]. Thus, time-dependent viscoelastic properties were assigned to the cytoplasm using this model (E_0_ = 6.5 kPa, E_∞_ = 4.3 kPa, ν = 0.5, τ = 15.4 s), where E_0_ is the instantaneous elastic modulus, E_∞_ is the equilibrium elastic modulus, ν is Poisson’s ratio, and τ is the characteristic relaxation time. Overall, the selected parameters are representative of an osteoblast in a “spread” morphology [76]. Isotropic, linear elasticity was prescribed for the cell nucleus with an elastic modulus four times stiffer than the instantaneous elastic modulus of the cytoplasm [81] and ν = 0.5. 

### 2.3. Effect of Viscoelasticity

To investigate the importance of viscoelasticity in the model, additional simulations were performed with the time constant τ assigned to the cytoplasm set to 0.1, 1.0, and 10 s (reflecting the wide range of values reported from experiments with atomic force microscopy and magnetic beads). Modeling was also performed in which the cytoplasm was assumed to be a linear elastic material, with elastic modulus of 6.5 kPa and Poisson’s ratio of 0.5. To investigate the importance of the differential elasticity of the cytoplasm and nucleus, an additional numerical simulation was also performed where the nucleus was assigned the same linear elastic modulus and Poisson’s ratio as the cytoplasm (*i.e.*, 6.5 kPa and 0.5). 

### 2.4. Effect of Cytoskeleton

**Figure 2:**
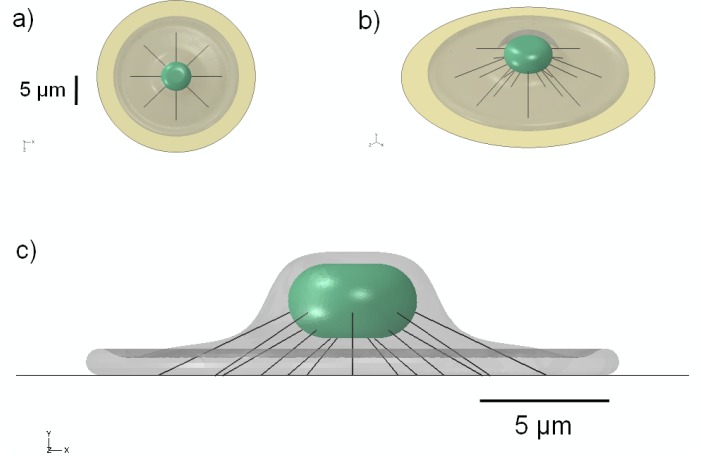
Schematic representation of the 3D model geometry used to represent a network of stress fibers, shown in: (**a**) top; (**b**) oblique; and (**c**) side view.

The nucleus in adherent cells is not rigidly embedded within the cytoplasm, but is more likely “tethered” to some extent by a network of actin stress fibers that connect the nucleus to the foot of the cell. To investigate the effect of a simple model of the cytoskeleton, a pre-tensioned cable network of stress fibers was used to model the actin cytoskeleton of the cell, similar in geometric configuration to that in [82]. Each actin stress fiber (SF) was represented as a linearly elastic cable supporting only tensile loads as described in [83]. To create the network, eight groups of three SFs were distributed radially at uniform 45-degree intervals for a total of 24 stress fibers (Figure 2). The SFs were discretized using non-compression bearing, 3-node quadratic truss elements for the finite-element simulations. Nodes at the free ends of the SFs were pin-joint constrained to allow for free rotation, with translational degrees of freedom constrained to those of the attachment surfaces. Values assigned for the elastic modulus and SF diameter were 1.45 MPa [83] and 0.3 μm [84], respectively. Pre-tensioning was accomplished through force-prescription, equivalent to 0.1% strain (1,000 μstrain) in each SF, which matched the driving radial strain prescribed on the elastomeric substrate membrane.

### 2.5. Finite-Element Modeling

Finite-element modeling was performed using a commercial FE software package (Abaqus CAE v.6.8.3, Simulia, Providence, RI, USA) running on a desktop computer (Intel Core i7 Quad, 4 cores, 2.67 GHz, maximum memory requirements 3 GBytes RAM). The model was discretized using 10-node solid 3D tetrahedral elements for the cell cytoplasm and nucleus, while 3-node planer 3D shell elements were employed for the elastomeric substrate membrane. The total number of elements was 58,656. The model was investigated at strain frequencies of 1, 20, and 45 Hz and computational simulation was carried out at each frequency for 10 complete input strain cycles. Data were recorded at 12 uniformly spaced time intervals per cycle for a total of 120 frames per frequency case. Each finite-element simulation required approximately 2 hours of run time on 2 CPU cores, producing three-dimensional maps of Von Mises stress, strain, and deformation. These quantitative maps were used to determine the maximum values of stress and strain over the strain cycle. The FEM package also produced time-resolved 3D cine loops of local stress and strain over the motion cycle; these movies were used to determine the location of stress concentrations within the cell.

## 3. Results and Discussion

### 3.1. 3D Model Geometry

The three-dimensional geometry of the simple cell model is shown in Figure 1; the shape that we have employed is similar to the “fried-egg” model of an attached cell *in vitro* [85]. As shown in the schematic, the tension on the underlying substrate is applied radially to produce an isotropic strain field under the attached cell. The cross-sectional image illustrates the location of the nucleus within the cytoplasm. The addition of stress fibers (Figure 2) mimics the configuration proposed in [82].

### 3.2. Von Mises Stress Distribution

**Figure 3:**
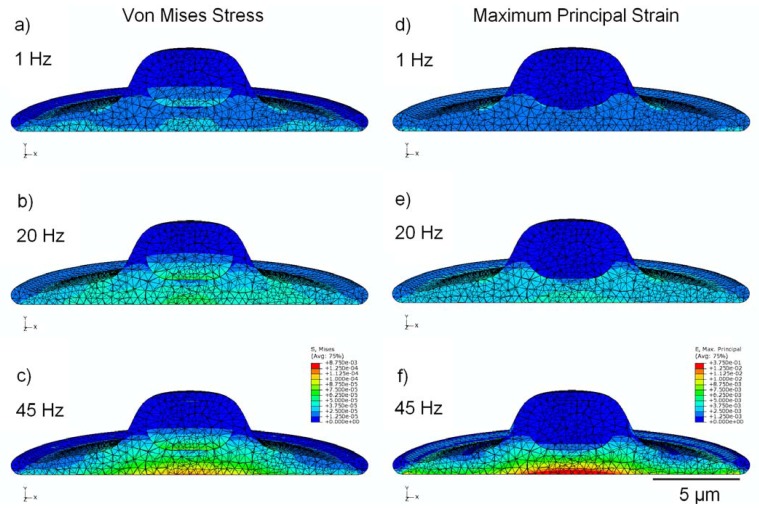
finite-element modeling (FEM) simulation results, showing peak values of stress in MPa (**a**,**b**,**c**) and strain (**d**,**e**,**f**) over the stimulation cycle, for frequencies of 1 Hz, 20 Hz, and 45 Hz. Cytoplasm is assumed to be viscoelastic, with the elastic modulus of the nucleus four times stiffer.

The stress distribution observed within the viscoelastic cell model is shown in Figure 3 (a,b,c), with cross-sectional results displayed for cyclic strain at 1, 20, and 45 Hz. Stress is noticeably greater in the model undergoing high-frequency stimulation, with peak stress values of around 87 Pa in the nucleus and 118 Pa in the cytoplasm; these values are approximately 130% and 320% higher in their respective regions at 45 Hz than at 1 Hz.

### 3.3. Strain Distribution

Figure 3 (d,e,f) shows the cross-sectional distribution of strain within the cell, under conditions of cyclic applied strain of 1,000 µstrain (*i.e.*, 0.1%). With 1 Hz applied strain, the maximum internal value of strain reported was 760 µstrain in the nucleus and 2,600 μstrain in the cytoplasm; whereas, at 45 Hz applied strain, the maximum value of internal strain over the stimulation cycle is 2,100 µstrain in the nucleus and 14,500 μstrain in the cytoplasm (increases of 175% and 450% in the nucleus and cytoplasm regions, respectively). Elevated values of strain were observed near the interface between the cytoplasm and the nucleus, possibly due to the dissimilar mechanical properties of these two components.

### 3.4. Effect of Viscoelasticity

**Figure 4:**
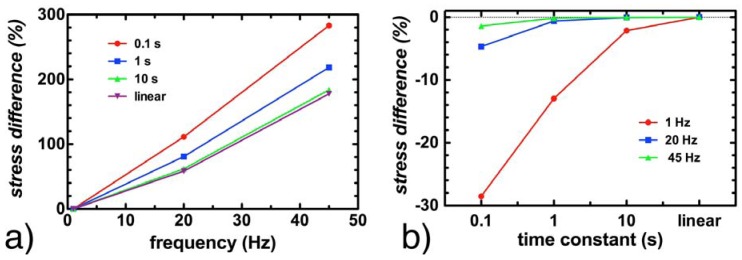
The impact of the assumed viscoelastic relaxation time constant τ at various frequencies. (**a**) shows the percentage increase in maximum cytoplasmic stress as a function of frequency, for τ = 0.1, 1 and 10 s, as well as for a linear elastic material; (**b**) shows the decrease in observed maximum cytoplasmic stress at 1, 20 and 45 Hz as a function of viscoelastic time constant, where the difference is with respect to a linear elastic model.

Simulations repeated with the time constant τ for the cytoplasm ranging from 0.1 to 10 s showed no impact on the strain values reported within the cell, regardless of the frequency of stimulation. Peak stress values were maintained within 5% at 20 Hz and 45 Hz, regardless of whether τ was set to 0.1 s, 1 s, 10 s or if the cytoplasm was assumed to be linearly elastic. At 1 Hz, peak stress values remained below 2.5% for τ = 10 s and when the cytoplasm was assumed to be linearly elastic. The only significant impact of modeling the cytoplasm as a viscoelastic material was observed with τ = 0.1 s and τ = 1 s for stimulation at 1 Hz; in these cases, the peak stresses reported in the cytoplasm were 28% and 13% lower respectively in a viscoelastic model than in a linear elastic model (Figure 4).

**Figure 5:**
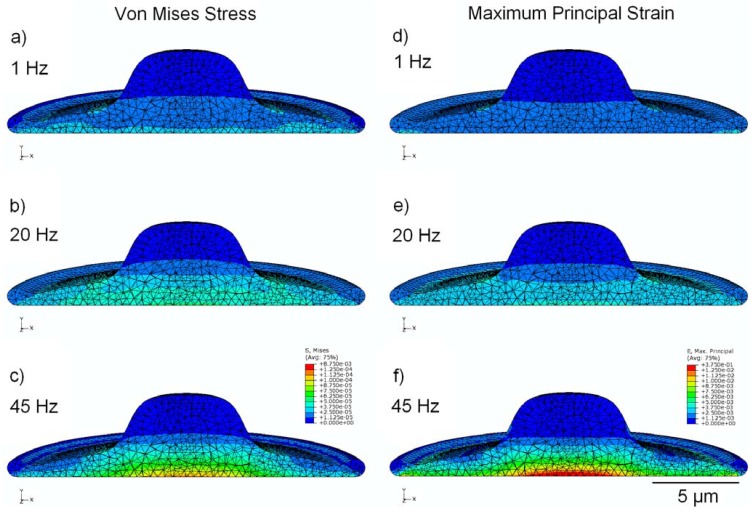
FEM simulation results under the assumption that both cytoplasm and nucleus are linear isoelastic materials, with elastic modulus of 6.5 kPa and Poisson’s ratio 0.5, showing peak values of stress in MPa (**a**,**b**,**c**) and strain (**d**,**e**,**f**) over the stimulation cycle, for frequencies of 1 Hz, 20 Hz, and 45 Hz.

The finite element model was repeated with both the nucleus and cytoplasm assumed to be linear elastic materials with identical elastic properties. In this case (Figure 5), maximum values of stress in the cytoplasm remained within 8% for all frequencies, however maximum stress in the nucleus was considerably reduced by approximately 50% in all cases with peak values of approximately 40 Pa, in comparison to peak stress of about 87 Pa when the nucleus was modeled as four times stiffer than the cytoplasm (Figure 3).

### 3.5. Effect of Cytoskeleton

**Figure 6:**
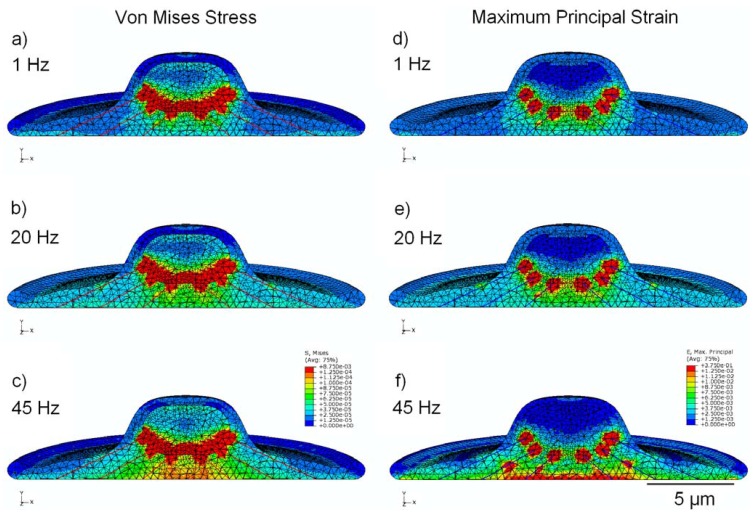
FEM simulation results, showing peak values of stress in MPa (**a**,**b**,**c**) and strain (**d**,**e**,**f**) over the stimulation cycle, for frequencies of 1 Hz, 20 Hz, and 45 Hz. This model incorporates cytoskeletal elements, represented by 24 pre-tensioned stress fibers.

Simulations performed with a model that incorporates a simplified cytoskeleton (Figure 6) showed significant focal stress concentrations at points where the pre-tensioned stress fibers attach to the peri-nuclear surface. Localized stress and strain values in the cytoplasm at the attachment points were found to be 1.5 kPa and 354,000 µstrain, respectively. Similarly, focal stress and strain values in the nucleus at the SF attachment points were 8.6 kPa and 279,000 µstrain, respectively. The maximum values for stress and strain at the attachment points—for both the nucleus and cytoplasm—were found to remain relatively constant (less than 1% variation) across all frequencies. To provide further insight into changes occurring in models containing the cytoskeleton and to make comparisons at different frequencies (excluding the singular and frequency-independent values at the attachment points), secondary areas of high stress and strain, distal to the SF attachments, were also analyzed. At 1 Hz, peak nuclear stress in these regions increased to 330 Pa in the cytoskeletal model, compared with 38 Pa in the viscoelastic model without cytoskeleton. Similarly, at 45 Hz peak nuclear strain increased to 7,900 µstrain in the cytoskeletal model, compared with 2,100 µstrain in the viscoelastic model of Figure 3. Stress values observed in the cytoplasm increased with frequency in this model, with peak stress increasing from 45 Pa at 1 Hz to 110 Pa at 45 Hz, representing a 150% increase. Peak strain in the cytoplasm also increased with frequency, with peak strain increasing from 3,700 µstrain at 1 Hz to 14,000 µstrain at 45 Hz, representing a 280% increase. Peak stress and strain in the nucleus both varied by less than 13% as a function of frequency, with average values of 350 Pa and 7,450 µstrain, respectively.

## 4. Conclusions

We have implemented the first finite-element model of a cell undergoing high-frequency equibiaxial strain on an elastomeric substrate. Moreover, we have used this model to determine the influence that the frequency of stimulation may play with respect to local stress and strain within the cell. Our results indicate that high-frequency stimulation (*i.e.*, 45 Hz oscillations) at 1,000 µstrain can introduce significantly elevated non-uniform strain inside the cell, with peak values of over 14,000 µstrain observed in the cytoplasm. This focal strain amplification was not observed at lower frequencies (*i.e.*, 1 Hz), but was observed in both linear elastic and viscoelastic models of the cell. Internal stress and strain values tend to concentrate near the interface between the cytoplasm and the nucleus, indicating the importance of the differential material properties of cellular constituents. Peak Von Mises stress values recorded at 45 Hz were more than twice as high in the nucleus and over four times higher in the cytoplasm than those observed at 1 Hz. These results may have implications in future studies aimed at understanding the role of high-frequency mechanical stimulation in mechanotransduction, suggesting that experiments and simulations should be carried out at a range of frequencies extending well above 1 Hz.

The inclusion of a simplified cytoskeleton, consisting of 24 stress fibers, produced focal elevations of stress and strain in the peri-nuclear region of the viscoelastic cell model. Focal values observed at the SF attachment points were highly elevated and were not strongly dependent on the frequency of stimulation; however, it is important to note that these values are reported at singularities and may be artifactually amplified. Representative regions of the cytoplasm (distal to the SF attachment points) exhibited frequency-dependent increases in both stress and strain. Our observations support the hypothesis that the cytoskeleton plays an important role in transmitting extracellular mechanical forces to the nucleus, possibly mediating mechanotransduction [86].

The value of the time constant τ that was assumed in the viscoelastic model did not have a significant impact on the derived stress and strain distributions, with the exception of the model simulating the two lowest time constants (0.1 s and 1 s) and lowest frequency (1 Hz). Under most conditions, at frequencies above 1 Hz, values of τ ranging from 1–15 s will provide acceptable results, as would a purely linear elastic model. Stress concentration was, however, influenced by the differential material properties in the model, such as the assumed mismatch between the properties of the nucleus and cytoplasm. These findings may indicate that in future models, consideration should be given to assigning accurate values to the elastic modulus of the different cellular components, as well as to the determination of viscoelastic parameters.

There are some simplifications in the finite-element model reported here. Adhesion was assumed to be uniform over the entire contact area between cell and substrate; in future studies it will be interesting to model the impact of focal adhesions. The axisymmetric three-dimensional geometry of the cell was not intended to represent *in vivo* conditions or the interactions of a cell with surrounding cells or the extracellular matrix; nonetheless, it is appropriate for comparison with previous and future *in vitro* experiments investigating equibiaxial strain with adherent monolayers [43,44,87]. The primary goal of this study was to investigate the role of frequency of stimulation, with all other factors held constant; in this context, the sophistication of the model was appropriate. Viscoelasticity within the cytoplasm was included in this model, although it was only found to be significant at the lowest frequency, or in general, in cases where the time constant is on the same order of, or smaller than, the period of the loading cycle. Otherwise, it was not found to be factor over the upper range of frequencies studied. The cytoskeletal model was necessarily simplistic, comprising only 24 stress fibers. Nonetheless, the inclusion of this simple cytoskeleton was shown to have a significant effect on focal stress and strain near the nucleus.

This study indicates the significant influence of the frequency of applied mechanical stimulation, in the case of equibiaxial strain at physiological levels. The elevated levels of stress and strain observed within the cytoplasm and nucleus may indicate a mechanism by which high-frequency mechanical stretch stimulates a response within cells. Future studies will include additional details of internal cytoskeletal structure and investigations of possible resonance conditions within the cell. The model will also be extended to include other mechanisms of cyclic stimulation (such as vibration and shear). Dynamic finite-element models will undoubtedly play an increasingly important role in investigating the way in which cells interact with biomaterial substrates and the local mechanical environment. Such models will also assist in elucidating the mechanisms underlying mechanotransduction. 
